# Myofunctional Trainer versus Twin Block in Developing Class II Division I Malocclusion: A Randomized Comparative Clinical Trial

**DOI:** 10.3390/dj8020044

**Published:** 2020-05-07

**Authors:** Yasmine Elhamouly, Azza A. El-Housseiny, Hanan A. Ismail, Laila M. El Habashy

**Affiliations:** 1 Pediatric Dentistry and Oral Public Health Department, Faculty of Dentistry, Alexandria University, Alexandria 21526, Egypt; yasmine.elhamouly@pua.edu.eg (Y.E.); azza.elhousseiny@dent.alex.edu.eg (A.A.E.-H.); 2 Orthodontic Department, Faculty of Dentistry, Alexandria University, Alexandria 21526, Egypt; hananaminismail@gmail.com

**Keywords:** myofunctional appliances, T4K, functional appliances, twin block, developing class II

## Abstract

This study aimed to evaluate and compare the dentoalveolar effects of the myofunctional trainer T4K^TM^ versus twin block in children with class II division I malocclusion. Two parallel arm randomized comparative clinical trial was conducted, including twenty healthy children, 9–12 years old, showing Angle’s class II division I malocclusion due to mandibular retrusion. Children were randomly assigned into two groups according to the appliance used; Group 1: T4k, and Group II: twin block. Follow-up was done every 4 weeks for 9 months. Postoperative cephalometric X ray, study casts and photographs were taken for measurements and comparison. T4K showed a statistically significant reduction in the overjet (−2.50 ± 1.00 mm) (*p* < 0.0001), and a significant increase in the lower arch perimeter (LAP) (1.19 ± 0.96 mm) (*p* = 0.01). The twin block showed a statistically significant reduction in the overjet (−3.75 ± 1.10 mm) (*p* < 0.0001), a significant reduction in the overbite (−16.22 ± 17.02 %) (*p* = 0.03), and a significant increase in the LAP (1.69 ± 0.70 mm) (*p* < 0.0001). The overjet showed a higher significant decrease in the twin block group than in T4K (*p* = 0.03). The mean values of the overbite were significantly decreased in twin block than in T4k (*p* < 0.0001). Both groups showed significant dentoalveolar improvements toward class I occlusion; however, the twin block showed significantly better results than T4K appliance.

## 1. Introduction

Developing class II malocclusion is one of the prevalent and dramatic problems in the mixed dentition stage, which demands early interception to decrease the severity of malocclusion, and thus the complexity and time of further orthodontic treatment [1]. Functional/myofunctional appliances are based on using muscle action to redirect dentoalveolar and skeletal growth toward normal occlusion. Isola et al. 2018 [2] pointed out the importance of adaptive response of the muscle fibers on the functional significance of masticatory muscles which influences the craniofacial characteristics, and malocclusion and consequently the treatment outcome. Moreover, in a later study in 2019, they highlighted the importance of healthy temporomandibular joint (TMJ) on the oral musculature and consequently on the quality of life [3]. It was found that functional appliances used in treatment of class II division I did not produce any significant adverse effects on the TMJ in healthy patients, the appliances also improved joints that initially presented with forward dislocation of the disk [4]. 

Many functional appliances were designed to work together with the natural growth pattern to enhance the mandible growth in a downward and forward direction in developing class II with mandibular deficiency [5]. The twin block is one of the most popular functional appliances used in treatment of developing class II. The appliance was developed by William J Clark [6] and has shown clinically successful improvements of the sagittal discrepancy in 6–9 months [7]. The appliance was also described to produce a dentoalveolar effect by enhancing differential eruption of the posterior teeth, resulting in an increased facial height, palatal tipping of the maxillary incisors and labial tipping of the mandibular incisors [7,8,9,10,11]. 

Myofunctional appliances in orthodontics are appliances that use muscle action to achieve the desired treatment. Pre-Orthodontic *Trainer for Kids*™ (T4K^TM^, Myofunctional research Co., Australia) was first introduced in 1992, and has become one of the most popular and successful products in guiding tooth eruption and correcting myofunctional habits in the early mixed dentition [12]. Tooth channels and labial bowls guide the erupting/developing dentition into correct alignment, while the tongue tag and lip bumpers treat myofunctional habits. It has a two phase treatment: phase one is a blue soft silicone appliance while phase two is a harder and stiffer polyurethane (pink or red in color). This was based on the same principle of orthodontic arch wire; “as the teeth come into place, more force can be used to encourage their alignment” [12]. T4K requires no impressions, no models, and can be used in children from 6 years of age with minimal chair time. The appliance must be worn for “1 h each day and overnight while sleeping”. The period of treatment usually requires 6 to 12 months from the beginning of phase two, using beyond this period is recommended, depending on the outcome and the next phase of orthodontic treatment [12]. 

Usumez et al., [13] demonstrated a significant overjet reduction, due to retroclination of the maxillary incisors and the significant proclination of the mandibular incisors, in their clinical study to evaluate the effects of early treatment with T4K on class II division I malocclusion on 20 children with an age range of 8.3–10.9 years old, compared to untreated controls with an age range of 9.6–11.0 years old. These findings were also supported by Dass and Reddy [14], who evaluated cephalometrically the treatment effects of the T4K on the dental and skeletal components in class II division I malocclusion on 20 patients of 8–12 years old for an observation period of 15 months. They noted a significant increase in the vertical dimension, as well as a reduction in the overjet, due to proclination of the mandibular incisors, and an increase in the mandibular incisor angle to the NB line in the treatment group over the untreated controls.

It has been demonstrated that individuals’ quality of life is related to their facial appearance, in which unpleasant esthetics due to malocclusion induces dissatisfaction, hinders professional achievements, emotional and social wellbeing, and negatively affects their self-esteem [15]. Body image starts to become an important feature of the child’s life at 6 years and gradually increases to reach its peak in the adolescence stage. Lack of acceptance and teasing by peers result in the frustration and emotional damage [16]. Starting treatment as early as possible is of great importance to improve the self-image in a growing child. Therefore, the role of pediatric dentists is crucial in guiding the dentoalveolar development and improving the self-image as early as possible, which was one of the vital incentives for conducting this work.

Most of the studies in the literature compared the T4K to untreated controls and rarely compared its effect to other functional appliances. Since dealing with children needs a lot of effort from the pediatric dentist, and the parents to convince the child to use these removable appliances, thus, a critical decision should be taken in deciding which appliance would provide the best treatment outcome within a shorter period of time. Therefore, the present study aimed to compare the dentoalveolar effects of T4K versus twin block in children with class II division I malocclusion. 

The null-hypothesis of this study was that there will be no significant difference in the dentoalveolar effects between the T4K and twin block at the end of the study.

## 2. Materials and Methods 

***Study Design:*** The study was two parallel arms randomized comparative clinical trial. This study was conducted over two-year period. This report was written according to the Consolidated Standard of Reporting Trials (CONSORT) statement [17]. 

***Participants:*** Twenty children were selected from the outpatient clinics of Pediatric Dentistry and Orthodontic Departments, Faculty of Dentistry, Alexandria University.

***Eligibility Criteria:*** Patients recruited were healthy children age ranged from 9–12 years, showing Angle’s class II division I malocclusion due to mandibular retrusion, depending on clinical judgement and confirmed with the lateral cephalometric X-ray. Patients with mandibular shifts, severe crowding, anterior open bite, posterior crossbite, any peri-oral habits or those who had received any previous orthodontic or orthopaedic treatments were excluded. Parental written consent was taken before treatment after explaining all the procedures, and for photographs taken of their children to be used in publication without any personal identification.

***Blinding:*** Two outcome assessors were blind to the appliance used during their evaluations of the X rays and the study models. Additionally, the statistician was blind to the appliances used in the group analysis.

***Randomization and Allocation Concealment:*** The twenty children were randomly assigned in a 1:1 ratio using a computer-generated list of random numbers [18] to one of the two groups: Group I, T4K group (pre-fabricated functional appliance, Myofunctional Research Co., Queensland, Australia). Group II, twin block group. An opaque envelope that was opened after seating the participant was used for allocation.

Ethics approval was taken from the Ethical Research Committee, Faculty of Dentistry, Alexandria University (IRB 00010556)-(IORG 0008839) on 17 April 2008. Trial identifier: NCT04337086.

***Sample Size Calculation***: Sample size was estimated based on the following assumptions: alpha error = 5%, study power = 80%. mean ± SD overbite change after using twin block appliance = 6.31 ± 1.71 [5] and was = 3.76 ± 1.60 when the T4k appliance was used [13]. Based on comparison of means, using the largest standard deviation to ensure study power, sample size was calculated to be 8 per group, which was increased to 10 to make up for cases lost to follow-up. The total sample size = number of groups × number per group = 10 × 2 = 20 [19].

**Figure 1:**
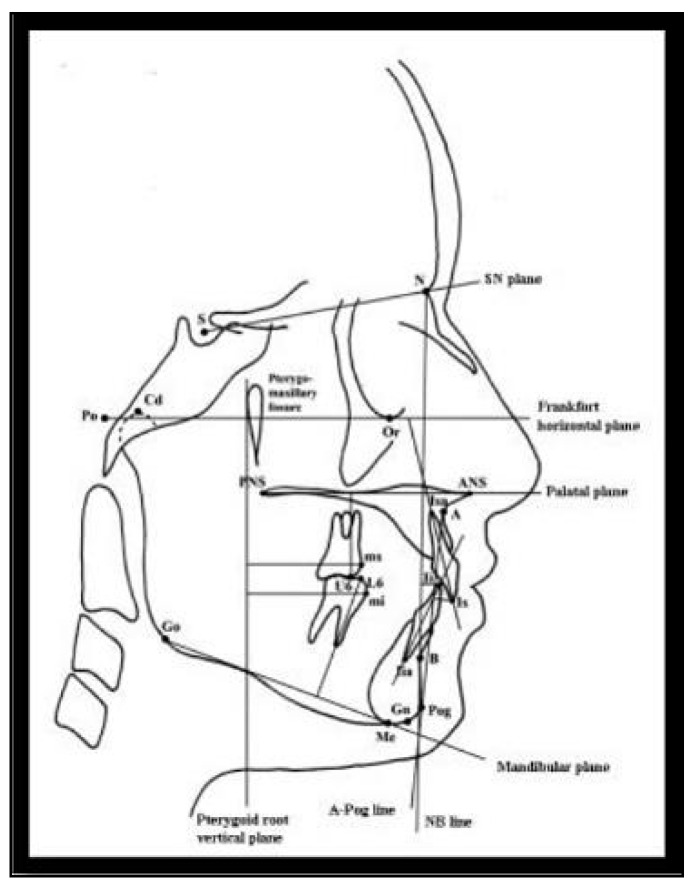
Photograph showing the reference points, angular and linear cephalometric measurements.

***Preoperative Records*:** A standardized lateral cephalometric X-ray was taken to determine the reference points, planes, angular and linear measurements to aid in diagnosis (Figure 1), and to act as a baseline record to monitor the dentoalveolar changes that occurred as a result of using the appliances. Orthopantomogram was also taken to check for any dental or bony abnormalities, state of shedding of the primary teeth, and eruption of the successors. Study casts were done using alginate impressions (Tulip, elastic high consistency, Cavex Holland BV) for the upper and lower arches and poured in the white stone (Timberlit, Protechno, Spain). Measurements obtained from the study cast included the following: overjet, overbite, molar relation and arch perimeter. Molar and canine relations were also determined and by clinical assessment. Extra-oral photographs (frontal, lateral views), and intra-oral photographs (frontal, lateral, occlusal views) were also obtained to serve as a base line data. Threshold values were: overjet greater than 3 mm, SNA angle in the range of 77–82.5 degrees, SNB angle in the range of 71–75 degrees and a Frankfort mandibular plane angle less than 30 degrees.

***Twin Block Construction*:** The customized twin block used was a modification of the Clark`s design [11] without midline screw. It consisted of Adams clasps, ball ended clasps and a maxillary labial bow with a 0.7 mm wire. The appliance was fabricated after a symmetric protrusive bite registration using pink wax sheets (Cavex set up regular modeling wax, Cavex Holland BV), in which the patients were rehearsed many times before its registration. They were asked to protrude the mandible symmetrically to an edge to edge relation and the midline was adjusted and marked. The casts were mounted on a simple hinge articulator using the wax bite with a clearance of 6–7 mm (indicating the vertical opening) in the buccal inter-occlusion, to permit adequate thickness for construction of the occlusal bite blocks [10]. Self-cure acrylic resin was used to construct the maxillary and mandibular acrylic plates and the active components of the appliance. The maxillary and mandibular inclined planes interlocked at 70 degrees to the occlusal plane mesial to the maxillary and the mandibular permanent molars in the region of the second premolar or the second primary molar, to reposition the mandible into an edge to edge relation. The mandibular bite block covered the mandibular premolars or the primary molars. The maxillary flat occlusal bite block passed distally over the rest of the maxillary posterior teeth, touching the mandibular permanent molars [6]. 

***Intervention***

***In Group I*:** The pre-fabricated, hard, polyurethane, pink T4K™ (Myo functional research Co., Australia) was used in the study. It consisted of facial bows, lingual bows/tongue guard, lingual tag/tongue tag, dental channels with a pre-determined bite position and a lip bumper (Figure 3). In the first week, the patients were instructed to gradually increase the wearing time of the appliance during the daytime. By the start of the second week, the patients were instructed to wear the appliance achieving at least 8 h during sleeping. The Trainer usually needs no adjustments. The patients were asked to place it themselves inside their mouths. The distal ends of the appliance were trimmed 2–3 mm with an acrylic bur on a straight hand piece if they were too long, or if the patient could not keep his lips together [12]. By the end of the first 4 weeks, the patients were wearing the appliances minimum of 1 h during the day plus the overnight wear following the manufacturer`s instructions. 

***In Group II*:** The twin block was used, and instructions were given to wear the appliance nearly fulltime. The patients were informed that they might experience some pain at the beginning of treatment, and that this pain will disappear as soon as they get used to the appliance. Selective sequential inter-occlusal trimming was done monthly after the second month in the occlusal surface of the maxillary bite blocks to clear over the mandibular molars, allowing their eruption in a more favorable mesial direction and to increase the vertical dimension. Trimming was done in the fitting surface over the maxillary and mandibular premolars whenever needed, to allow their full eruption and to level the occlusal plane [6]. The examination was carried out by two trained and calibrated examiners according to inter-rater agreement [20].

***Outcome Assessment:*** All patients were scheduled after one week to check for any complaints, follow-up visits were then done every 4 weeks for 9 months. After 9 months (active therapy), the final records were taken (lateral cephalometric X-rays, study casts, extra and intra oral photographs) and compared to the pre-intervention records. The appliances were then kept in their mouths for another 6 months for more retention and stability. Patients that needed further fixed orthodontic treatment were referred to the Orthodontic Department. 

***Statistical Analysis*:** Sample description as regards age (mean and standard deviation) and sex (number and percent) was compared using *t* test. Normality was checked and descriptive statistics were displayed as means and standard deviations for the different measurements. Comparison of the values of the measurements, before and after intervention in each group separately, was done using paired *t* test. Comparison of the values before and after intervention between the 2 groups was done using *t* test. Statistical analysis was done using IBM SPSS version 15.0. Significance was determined at 5% level of confidence, if *p* ≤ 0.05 it was considered significant [21].

## 3. Results

**Figure 2:**
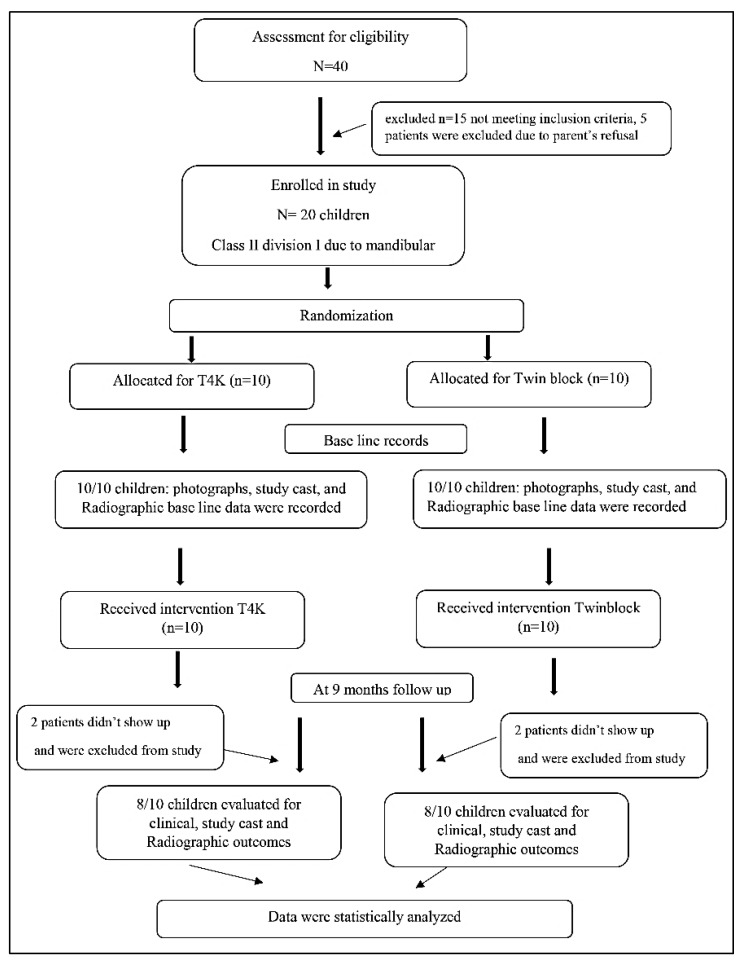
Consolidated Standard of Reporting Trials (CONSORT) diagram showing study protocol up to 9-month follow up.

The sample enrolled in this study was twenty patients divided equally among the two groups. Two patients were dropped out from each group (two patients refused the T4K, as they found it very inconvenient to wear, and the parents did not want to force their children to wear the appliance. In the twin block, two children refused to wear the appliance in school time, which is a proximally 8 h, because they were mocked by their peers) and the statistical analysis was done only for 16 patients with a mean age of 10.6 ± 0.6 years. Group I (T4K): included four females (50%) and four males (50%). Group II (twin block): included six females (75%) and two males (25%). The flow chart of the study is reported in Figure 2.

Table 1 shows there was no statistically significant difference in all the mean dentoalveolar angular or linear measurements or study cast measurements between the 2 groups, (*p* > 0.05) before intervention.

### 3.1. Cephalometric Dentoalveolar Changes after Appliance Therapy

**Figure 3:**
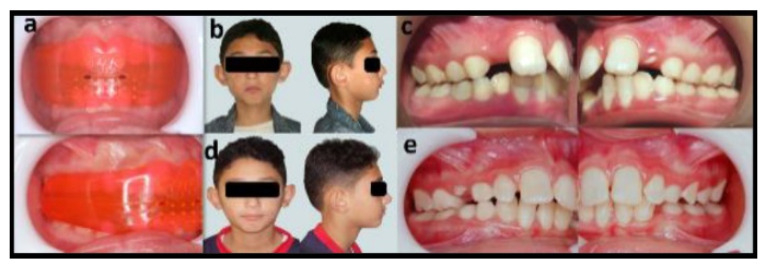
Photographs showing the T4K myofunctional appliance group: (**a**) intraoral frontal and lateral views of the appliance inside patient’s mouth; (**b**) preoperative frontal and lateral extraoral views showing a convex profile and incompetent lip seal; (**c**) preoperative right/left lateral intraoral views showing class II canine and molar relation; (**d**) postoperative frontal and lateral extraoral views showing a complete lip seal and straight facial profile; (**e**) postoperative right/left lateral intraoral views showing class I canine and molar relation and reduced overjet.

The mean dentoalveolar angular (degrees) and linear (mm) measurements, before and after intervention in Group I “T4K” (Table 2 and Figure 3).

Angular Measurements

There was statistically significant decrease in the axial inclination of the maxillary incisor to the Frankfort horizontal plane U1-FHP (*p* = 0.01), with a mean difference of −4.00 ± 2.98 degrees. There was statistically significant increase in the mean values of the axial inclination of the mandibular incisor to the mandibular plane L1-MP (*p* = 0.003), with mean differences of 1.94 ± 1.27 degrees. 

Linear Measurements

There was statistically significant decrease in the mean value of the position of the tip of the maxillary incisor in relation to the A-Pog Line Is-APog (*p* = 0.001), with a mean difference of −2.00 ± 1.04 mm. There was statistically significant increase in the mean value of the position of the tip of the mandibular incisor in relation to the NB line Ii- NB (*p* = 0.003), with a mean difference of (1.06 ± 0.68) mm. The vertical distances between the maxillary molar to the palatal plane U6-Pl.P, and the mandibular molar to the mandibular plane L6-Mp showed a statistically significant increase (*p* = 0.05) and (*p* = 0.01), with mean differences of 0.38 ± 0.44 and 0.50 ± 0.38 mm, respectively. The antero-posterior position of the mandibular and the maxillary first permanent molars in relation to the vertical reference line mi-PtV, and ms-PtV showed a statistically significant increase (*p* < 0.0001) and (*p* = 0.003), with mean differences of 1.75 ± 0.76 and 1.06 ± 0.68 mm, respectively.

**Figure 4:**
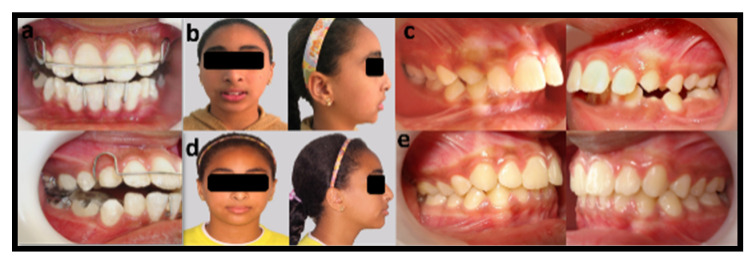
Photographs showing twin block appliance group: (**a**) intra oral frontal and lateral views of the appliance inside the patient’s mouth; (**b**) preoperative frontal and lateral extraoral views, showing a convex facial profile and incompetent lip seal; (**c**) preoperative right/left lateral intraoral view, showing class II canine and molar relation; (**d**) postoperative frontal and lateral extraoral views, showing a competent lip seal and straight facial profile; (**e**) postoperative right/left lateral intraoral views, showing class I canine and molar relation and reduced overjet.

The mean dentoalveolar angular (degrees) and linear (mm) measurements, before and after intervention in Group II “Twin block” (Table 3 and Figure 4).

Angular Measurements

There was statistically significant decrease in the U1-FHP angle (*p* = 0.001), with a mean difference of −7.75 ± 3.96 degrees. There was statistically significant increase in the mean values of the L1-MP (*p* = 0.001), with a mean difference of 2.19 ± 1.19 degrees. 

Linear Measurements 

There was statistically significant decrease in the mean value of Is-APog (*p* < 0.0001), with a mean difference of −3.00 ± 0.60 mm. There was statistically significant increase in the mean value of Ii-NB (*p* < 0.0001), with a mean difference of 1.19 ± 0.46 mm. The vertical distances U6-Pl.P, and L6-Mp showed statistically significant increases (*p* < 0.0001), with mean differences of 0.38 ± 0.44 and 2.13 ± 0.95 mm, respectively. The horizontal distance mi-PtV, showed a statistically significant increase (*p* < 0.0001), with a mean difference of 2.00 ± 0.60 mm. No significant difference was detected in the horizontal distance ms-PtV before and after intervention, (*p* > 0.05).

**Figure 5:**
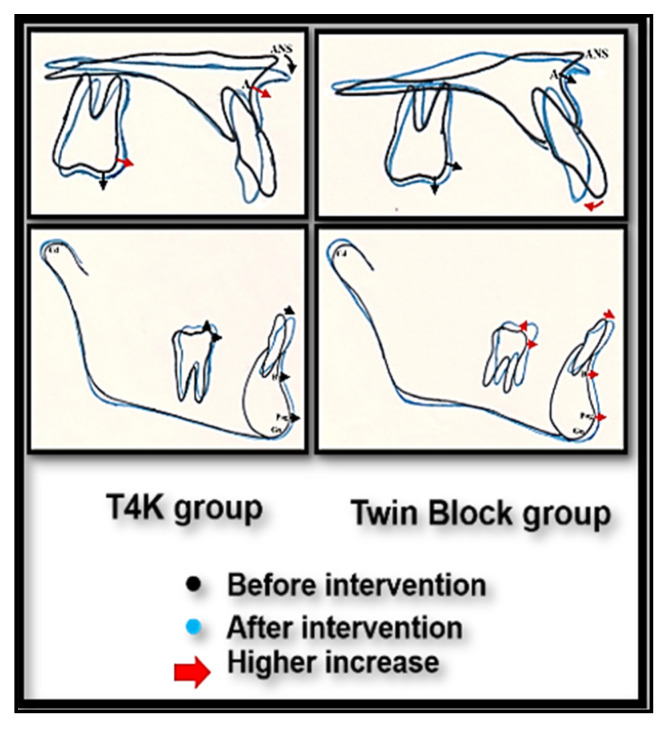
Photographs showing Superimposition of the maxilla and mandible before and after treatment in both the T4K and twin block groups. (Retroclination of the maxillary incisor, proclination of the mandibular incisor, mesial and vertical movement of the maxillary and mandibular permanent molars).

Table 4 and Figure 5 show the result of the appliance therapy in both groups. The mean value of the U1-FHP angle showed a significant decrease (*p* = 0.05) in the twin block group—more than that seen in the T4K group. There was significant decrease in the mean value of Is-APog (*p* = 0.04) in Group II than in Group I. There was significant increase in the horizontal distance ms-PtV (*p* = 0.03) in Group I than Group II. The vertical distance L6-MP significantly increased (*p* = 0.001) in Group II, compared to Group I. 

### 3.2. Study Cast Changes after Appliance Therapy

In Group I (T4K), the overjet was significantly reduced (*p* < 0.0001), with a mean difference of −2.50 ± 1.00 mm. The LAP showed a significant increase (*p* = 0.01), with a mean difference of 1.19 ± 0.96 mm. There was no statistically significant difference in the “overbite and UAP” before and after intervention, (*p* > 0.05), Table 2.

In Group II (twin block), the overjet and the overbite were significantly reduced by (*p* < 0.0001) and (*p* = 0.03), respectively, with mean differences of −3.75 ± 1.10 mm and −16.22 ± 17.02 respectively. No significant difference was detected in the mean values and the UAP before and after intervention, (*p* > 0.05), Table 3.

The overjet more significantly decreased in Group II than in Group I (*p* = 0.03). The mean values of the overbite, UAP and LAP were more significantly increased in Group I than in Group II, by (*p* = 0.007), (*p* = 0.02) and (*p* < 0.0001) respectively (Table 4). 

The improvement towards full class I molar relation was determined by a Z proportional test, where there was statistically significant difference (*p* = 0.038) in Group II (87.5%) compared to Group I (37.5%).

## 4. Discussion

The null hypothesis of this study was rejected, as there was a statistically significant difference between the two studied groups.

Improving self-image is of major significance in a growing child as this will directly affect their self-esteem, which is fundamental in building their personality and self-acceptance. Although self-image reaches its peak in adolescence, it actually starts earlier, at 6 years of age [15,16]. Hence, the pediatric dentist should interfere as early as possible with interceptive orthodontic measures to improve or correct any detected malocclusion that could alter the child’s quality of life. Moreover, it should be taken into consideration that the success and stability of the treatment outcomes are heavily dependent on the oral and masticatory muscles adaptive responses [2] and the pressure equilibrium between the tongue, lips, and cheeks [22].

Prefabricated myofunctional appliances has gained popularity in correcting various types of malocclusion, including developing class II malocclusion. They target the etiological factors leading to malocclusion by correcting the dysfunction in the orofacial muscle activity, the tongue posture and improving the airway volume [22,23], resulting in a more stable occlusion. However, their effectiveness is considered a controversial topic, with conflicting debates and opinions. Therefore, this study aimed to compare the effectiveness of myo-functional appliance “T4K” versus the functional appliance “twin block” on class II division I.

This study did not include untreated control group in order to differentiate the dentoalveolar treatment effects of both appliances from the natural growth, as this would raise an ethical problem. Therefore, the twin block appliance was used as a comparison group. 

The sample in the current study included patients with class II division I malocclusion due to mandibular retrusion, confirmed by the lateral cephalometric X-ray. Children selected in this study were between 9–12 years old, as most of the clinical trials data has proven that the maximum effect of the myofunctional/functional appliances is achieved during the prepubertal period of accelerated growth [5]. Owing to the small sample size and random allocation, the number of males and females was not equally distributed among the two groups; therefore, it was not feasible to compare the effects of the appliances between males and females. For standardization purposes, all selected cases enrolled in this study showed no statistically significant difference in the base line linear and angular measurements between the two study groups.

In this study, the hard, polyurethane (red/pink) second phase “T4K” appliance was used, as patients with perioral habits who required phase I treatment (soft, blue silicone appliance) were excluded from the study sample. The twin block design used in this study was adopted from Khafagy [11] in three modifications to the Clark’s twin block [6]. Firstly, the maxillary midline expansion screw was not incorporated in the appliance design. The reason for this was because patients with improper maxillary width or showing a cross bite when the mandible was advanced in the clinical examination were not included in the study sample. Secondly, Adams clasps were placed on the maxillary and mandibular permanent molars instead of primary molars. This was because the study was performed on children with mixed dentition, primary molars might shed during the treatment period, and their successors might not have been erupted yet. Finally, Ball ended clasps placed on the mandibular incisors were used to aid in retention in this young age.

The Overjet changes were determined from three lines of measurements (angular cephalometric changes, linear cephalometric changes, and study cast measurement).

The angular changes of the present study showed that both appliances significantly decreased axial inclination of the maxillary incisor to the Frankfort horizontal plane (U1-FH), and significantly increased the axial inclination of the mandibular incisor to the mandibular plane (L1-MP), reflecting an overjet reduction.

The linear changes observed in both groups were in parallel arm, with the angular changes suggesting a significant alternation toward favorable teeth alignment. Both study groups showed statistically significant decrease in the position of the tip of the maxillary incisor (Is) in relation to the A-Pog Line (Is-APog), with a significant increase in the mean value of the position of the tip of the mandibular incisor (Ii) in relation to the NB line (Ii-NB). Moreover, these cephalometric findings were confirmed by the cast measurements’ changes where the overjet showed a mean decrease of (−2.50 ± 1.00 and −3.75 ± 1.10) in the T4K and twin block groups respectively. 

The overjet reduction encountered by both appliances in this study was supported by many other studies in the literature, where Usumez et al. [13], Dass and Reddy [14] reported a significant reduction in the overjet in children between 8–12 years over a mean period of 13 and 15 months, respectively, when using the T4K appliance compared to the untreated controls. Similar findings were reported for the twin block appliance [8,9,24]. It should be noted that, although both appliances showed significant reduction in the overjet, the twin block showed higher performance, with significantly better results in the angular, linear and cast measurements. The better performance of the twin block toward favorable teeth alignments, might be attributed to the stronger action by utilizing the resultant masticatory forces in redirecting the arches toward normal relation [8]. Whereas the T4k depended mainly on modifying the oral musculature acting on the dental arches [25], which might have needed more time to produce its desirable effect. 

The permanent molar relation changes were determined by linear cephalometric changes and study cast measurements. The sagittal relation of the mandibular (mi) and the maxillary (ms) first permanent molars to the vertical reference line (mi-PtV) and (ms-PtV) showed a statistically significant increase in both groups. However, the mandibular molars showed more mesial movement than maxillary molar favoring class I relation. These findings were in agreement with other studies, whether for the T4K [13,14] or twin block appliance [5,11]. The justification for molar relation improvement was based on the action of both appliances that share the same concept of forcing the patient to close in class I relation, resulting in mandibular forward movement with slight maxillary restriction. In the present study, the twin block showed significant improvement in molar relation than the T4K appliance, where 87.5% of the twin block group, compared to only 37.5% of the T4K group, had reached full class I relationship, whereas 12.5% in the twin block group, compared to 62.5% in the T4K group, had reached an end to end relation. The justification for the better results in the twin block group could be clearly related to the selective sequential inter-occlusal trimming, which allowed the mandibular molar eruption in a mesial direction to correct the molar relation. On the other hand, this trimming was not feasible for the T4K group. Another important factor is the duration of the appliance use, which was nearly full time, except during eating, in the twin block, compared to the reduced time wear in the T4k appliance, which was only worn for 1–2 h during the day, in addition to during sleep, which was part of the T4K limitations. 

The overbite changes were determined by linear cephalometric changes and study cast measurements. It is well known that the overbite is in an inverse relation with the vertical molar relation. Since, in the present study, the vertical position of the maxillary molar to the palatal plane (U6-Pl.P) and the mandibular molar to the mandibular plane (L6-Mp) showed a statistically significant increase in the mean value of both groups. Therefore, this molars’ extrusion would indicate overbite reduction, this was obvious in the twin block group that showed a significant mean decrease in the overbite of (−16.22% ± 17.02%, and was in accordance with other studies [8,26,27]. On the other hand, the T4K appliance showed unexpected increase in the overbite of 4.65% ± 7.65%, and this finding was inconsistent with other previous studies [13,28,29], which reported the T4K reduced the bite by the extrusion of the maxillary and mandibular molars that accompanied their mesial movement. The explanation for the increased overbite with the T4K group in this study was attributed to the poor retention and looseness of the appliance, leaving a space between the teeth and the appliance [30]. As a result, extrusion of the maxillary incisors might have taken place and accounted for the overbite increase. 

Arch perimeter changes: the upper arch perimeter did not show any significant changes in both groups; however, the lower arch perimeter showed a significant increase in T4K group, and a significant decrease in the twin block group. The LAP increase with the T4K might be attributed to the lip bumper effect of the appliance, which released the tension of the mentalis muscle acting upon the dentition, allowing a significant proclination of the lower incisors and, accordingly, an increase the LAP. On the other hand, in the twin block group the significantly higher proclination and mesial movement of the mandibular permanent molars resulted in decrease of the LAP, despite the proclination that occurred in the lower incisors. 

The difference in appliances’ retention played a major role in this study out comes. The limitations of the T4K performance was in agreement with the clinical data obtained from recent systematic reviews [22,23,31], where the patients showed poor compliance due to looseness of the appliance, especially during sleep, leaving a space between the teeth and the appliance. Moreover, during the day, the T4K restricted their speech abilities and life activities. This was inconvenient to the children and took them a long time to accept the appliance, which accordingly delayed and hindered the appliance action [30]. On the other hand, the excellent retention of the twin block and the higher reciprocal force acting distally on the maxilla upon the nearly fulltime wear resulted in the significantly better outcomes. Furthermore, the twin block was more acceptable to the patients, as it did not hinder their speech and life activities.

Finally, it has to be noted that among the limitations of this study was the follow-up period, which was only nine months only and could have affected the T4k outcomes, as most of the children needed a couple of months to properly wear the appliance, achieving the required 8 h per day. To overcome this limitation, patients were followed up for another six months after the study period, to ensure the retentive period and overcome any relapses. At that stage, patients who needed further treatment were referred to the Orthodontic Department for fixed appliance therapy.

This study strength was based on comparing the T4k to another functional appliance to clearly demonstrate its limitations or advantages, which may not be well manifested if compared to an untreated control. This is especially important when dealing with children, where rapid treatment and the child’s compliance are important points of consideration.

The interest of this article might be limited to the orthodontists, as they might always choose the twin block for its better outcome, but this research was done mainly to address the pediatric dentists’ need to provide interceptive orthodontic treatment, according to their limited knowledge in that field compared to the orthodontist. As we believe that one of the major goals for pediatric dentists is to guide occlusion in a normal direction as much as possible, and since not all pediatric dentists can use the twin block, this study showed that the T4k (where the majority of pediatric dentists are well aware of the appliance and use it in habit breaking therapy) can help in minimizing the severity of the problem, so that when the child is 12 years old, the burden on the orthodontist to continue the treatment is much less. This is a major role between orthodontists and pedodontists to serve for the benefits of children with various malocclusions. 

## 5. Conclusions

Based on this short-term study, it was concluded that both T4k and twin block appliances achieved significant improvement in the anteroposterior inter-arch relationship in developing class II division I patients. The twin block appliance showed a significant improvement in the vertical inter-arch relationship with significant decreases in the overbite, but on the other hand the T4K appliance showed an increase in the overbite results. It has to be noted that the major limitation of the T4K was its poor retention and low patient compliance.

Accordingly, if the practitioner lacks the skill for the twin block appliance, as not all pediatric dentists are acquainted with it, or if the patient is a vertical grower with an increased facial height or his class II malocclusion is coupled with an oral habit or lower arch crowding, the best choice would be the use of the T4K, given that the patient and the parents are very co-operative. On the other hand, the twin block needs a skilled practitioner, increases the facial height, and has no significant effect on oral habits or lower arch widening. However, if the patient has a severely increased overjet, or cannot comply with the use of the one piece appliance, it is preferable to use the twin block, as the bite could be incrementally advanced to reach class I occlusion, and was more accepted by the patients, unlike the T4K, which is mounted to class I and showed lower compliance and acceptance.

## 6. Recommendations

Long term studies with larger sample size are still needed to evaluate the effect of T4K in enhancing dental arch measurements in the mixed dentition of Class II division I children, and the effect of gender on the appliance outcomes. It would be a point of interest to conduct further studies to evaluate the effect of myofunctional appliances in children with TMJ disorders as this would directly affect the muscles’ actions and, accordingly, the appliance outcomes.

## Figures and Tables

**Table 1:** Mean dentoalveolar angular (degrees) and linear measurements before intervention in Group I and Group II.

Measurements	Before Intervention Mean ± SD		*t*-Test *p* Value
	Group I (*N* = 8)	Group II (*N* = 8)	
**Cephalometric Angular Measurements**			
Upper incisor to Frankfort Horizontal plane (U1-FHP) (°)	119.81 ± 4.52	121.13 ± 4.22	0.60 0.56
Lower incisor to mandibular plane (L1-MP) (°)	95.50 ± 1.67	95.44 ± 3.41	0.05 0.96
**Cephalometric Linear Measurements**			
Maxillary incisor to APog line (Is-APog) (mm)	10.38 ± 2.26	11.13 ± 1.55	0.77 0.45
Mandibular incisor to NB line (Ii-NB) (mm)	5.19 ± 0.96	6.63 ± 1.58	2.20 0.06
Maxillary molar to palatal plane (U6-PL.P) (mm)	20.50 ± 1.77	19.25 ± 2.42	1.18 0.26
Mandibular molar to mandibular plane (L6-MP) (mm)	28.69 ± 1.53	27.44 ± 3.11	1.02 0.33
Mandibular molar to PtV line (mi-PtV) (mm)	22.13 ± 4.90	24.44 ± 4.99	0.94 0.37
Maxillary molar to PtV line (ms-PtV) (mm)	25.13 ± 3.93	27.06 ± 5.26	0.83 0.42
**Study Cast Measurements**			
Overjet (mm)	7.88 ± 1.66	7.94 ± 1.82	0.07 0.94
Overbite (%)	46.47 ± 23.54	61.39 ± 17.66	1.43 0.17
Upper Arch perimeter (UAP) (mm)	88.06 ± 4.44	83.00 ± 5.20	2.09 0.06
Lower Arch perimeter (LAP) (mm)	72.63 ± 2.94	72.56 ± 6.96	0.02 0.98

Statistically significant at *p* ≤ 0.05.

**Table 2:** Mean dentoalveolar measurements before and after intervention in Group I T4K.

Measurements	Before Intervention Mean ± SD	After Intervention Mean ± SD	Paired *t*-Test *p* Value	Difference Mean ± SD
**Cephalometric Angular Measurements**				
Upper incisor to Frankfort Horizontal plane (U1-FHP) (°)	119.81 ± 4.52	115.81 ± 5.47	3.80 0.01 *	−4.00 ± 2.98
Lower incisor to mandibular plane (L1-MP) (°)	95.50 ± 1.67	97.44 ± 1.70	4.33 0.003 *	1.94 ± 1.27
**Cephalometric Linear Measurements**				
Maxillary incisor to APog line (Is-APog) (mm)	10.38 ± 2.26	8.38 ± 2.63	5.47 0.001 *	−2.00 ± 1.04
Mandibular incisor to NB line (Ii-NB) (mm)	5.19 ± 0.96	6.25 ± 1.39	4.43 0.003 *	1.06 ± 0.68
Maxillary molar to palatal plane (U6-PL.P) (mm)	20.50 ± 1.77	20.88 ± 1.79	2.39 0.05 *	0.38 ± 0.44
Mandibular molar to mandibular plane (L6-MP) (mm)	28.69 ± 1.53	29.19 ± 1.36	3.74 0.01 *	0.50 ± 0.38
Mandibular molar to PtV line (mi-PtV) (mm)	22.13 ± 4.90	23.88 ± 4.49	6.55 <0.0001 *	1.75 ± 0.76
Maxillary molar to PtV line (ms-PtV) (mm)	25.13 ± 3.93	26.19 ± 3.89	4.43 0.003 *	1.06 ± 0.68
**Study Cast Measurements**				
Overjet (mm)	7.88 ± 1.66	5.38 ± 2.17	7.07 <0.0001 *	−2.50 ± 1.00
Overbite %	46.47 ± 23.54	51.12 ± 21.64	1.72 0.13	4.65 ± 7.65
Upper Arch perimeter (UAP) (mm)	88.06 ± 4.44	88.94 ± 4.84	1.67 0.14	0.88 ± 1.48
Lower Arch perimeter (LAP) (mm)	72.63 ± 2.94	73.81 ± 2.83	3.49 0.01 *	1.19 ± 0.96

* Statistically significant at *p* ≤ 0.05.

**Table 3:** Mean dentoalveolar measurements before and after intervention in Group II: twin block.

Measurements	Before Intervention Mean ± SD	After Intervention Mean ± SD	Paired *t*-Test *p* Value	Difference Mean ± SD
**Cephalometric Angular Measurements**				
Upper incisor to Frankfort Horizontal plane (U1-FHP) (°)	121.13 ± 4.22	113.38 ± 3.89	5.54 0.001 *	−7.75 ± 3.96
Lower incisor to mandibular plane (L1-MP) (°)	95.44 ± 3.41	97.63 ± 4.30	5.19 0.001 *	2.19 ± 1.19
**Cephalometric Linear Measurements**				
Maxillary incisor to APog line (Is-APog) (mm)	11.13 ± 1.55	8.13 ±1.27	14.20 <0.0001 *	−3.00 ± 0.60
Mandibular incisor to NB line (Ii-NB) (mm)	6.63 ± 1.58	7.81 ± 1.62	7.33 <0.0001 *	1.19 ± 0.46
Maxillary molar to palatal plane (U6-PL.P) (mm)	19.25 ± 2.42	19.63 ± 2.75	2.39 0.05 *	0.38 ± 0.44
Mandibular molar to mandibular plane (L6-MP) (mm)	27.44 ± 3.11	29.56 ± 3.27	6.30 <0.0001 *	2.13 ± 0.95
Mandibular molar to PtV line (mi-PtV) (mm)	24.44 ± 4.99	26.44 ± 5.21	9.47 <0.0001 *	2.00 ± 0.60
Maxillary molar to PtV line (ms-PtV) (mm)	27.06 ± 5.26	27.19 ± 5.62	0.40 0.70	0.13 ± 0.88
**Study Cast Measurements**				
Overjet (mm)	7.94 ± 1.82	4.19 ± 1.39	9.63 <0.0001 *	−3.75 ± 1.10
Overbite (%)	61.39 ± 17.66	45.17 ± 12.20	2.70 0.03 *	−16.22 ± 17.02
Upper Arch perimeter (UAP) (mm)	83.00 ± 5.20	81.75 ± 5.96	2.24 0.06	−1.25 ± 1.58
Lower Arch perimeter (LAP) (mm)	72.56 ± 6.96	70.88 ± 6.99	6.78 <0.0001 *	−1.69 ± 0.70

* Statistically significant at *p* ≤ 0.05.

**Table 4:** Mean dentoalveolar angular and linear measurements change after intervention between Group I and Group II.

Measurements	After Intervention Mean ± SD		*t*-Test *p* Value
	Group I (*N* = 8)	Group II (*N* = 8)	
**Cephalometric Angular Measurements**			
Upper incisor to Frankfort Horizontal plane (U1-FHP) (°)	−4.00 ± 2.98	−7.75 ± 3.96	2.14 0.05 *
Lower incisor to mandibular plane (L1-MP) (°)	1.94 ± 1.27	2.19 ± 1.19	0.41 0.69
**Cephalometric Linear Measurements**			
Maxillary incisor to APog line (Is-APog) (mm)	−2.00 ± 1.04	−3.00 ± 0.60	2.37 0.04 *
Mandibular incisor to NB line (Ii-NB) (mm)	1.06 ± 0.68	1.19 ± 0.46	0.43 0.67
Maxillary molar to palatal plane (U6-PL.P) (mm)	0.38 ± 0.44	0.38 ± 0.44	0 1.00
Mandibular molar to mandibular plane (L6-MP) (mm)	0.50 ± 0.38	2.13 ± 0.95	4.48 0.001 *
Mandibular molar to PtV line (mi-PtV) (mm)	1.75 ± 0.76	2.00 ± 0.60	0.73 0.48
Maxillary molar to PtV line (ms-PtV) (mm)	1.06 ± 0.68	0.13 ± 0.88	2.39 0.03 *
**Study Cast Measurements**			
Overjet (mm)	−2.50 ± 1.00	−3.75 ± 1.10	2.38 0.03 *
Overbite %	4.65 ± 7.65	−16.22 ± 17.02	3.16 0.007 *
Upper Arch perimeter (UAP) (mm)	0.88 ± 1.48	−1.25 ± 1.58	2.77 0.02 *
Lower Arch perimeter (LAP) (mm)	1.19 ± 0.96	−1.69 ± 0.70	6.83 <0.0001 *

* Statistically significant at *p* ≤ 0.05.
